# Sense of Coherence, Social Support, Maternal-Fetal Attachment, and Antenatal Mental Health: A Survey of Expecting Mothers in Urban India

**DOI:** 10.3389/fgwh.2021.714182

**Published:** 2021-09-07

**Authors:** Moksha Pasricha, Suhaavi Kochhar, Ashumi Shah, Avantika Bhatia

**Affiliations:** ^1^Department of Psychology, Ashoka University, Sonipat, India; ^2^Faculty of Social and Behavioral Sciences, University of Amsterdam, Amsterdam, Netherlands

**Keywords:** antenatal, depression, maternal-fetal attachment, pregnancy, prenatal, sense of coherence, social support, mental well-being

## Abstract

**Introduction:** Pregnancy is associated with psychological, physiological and social shifts, and can be a vulnerable time in a woman's life. Despite a growing understanding of the importance of antenatal mental health, there is a paucity of research on psychosocial factors relevant to this phase, especially in developing countries. The aim of the present study was to investigate the associations of expecting mothers' sense of coherence, perceived social support, and maternal-fetal attachment with mental health outcomes.

**Method:** Participants (*N* = 122) were nulliparous expectant mothers residing in urban India. Cross-sectional data was collected using an online questionnaire.

**Results:** Participant reports of perceived social support and sense of coherence were negatively correlated with symptoms of antenatal depression, while reports of maternal-fetal attachment, sense of coherence, and social support were positively associated with antenatal well-being. In a multilinear regression model, perceived social support and sense of coherence uniquely contributed to symptoms of antenatal depression, while maternal-fetal attachment and sense of coherence uniquely contributed to antenatal well-being.

**Discussion:** The findings of this study highlight the role of perceived social support, sense of coherence and maternal-fetal attachment in contributing to expecting mothers' mental health and well-being in urban India. These findings have implications for clinical practice and research, intending to the subjective experiences of pregnant women to improve antenatal mental health. Future research investigating these psychosocial factors using longitudinal designs is warranted and would help clinicians and practitioners identify women at risk for perinatal mental health concerns.

## Introduction

The period from conception to the birth of the first child marks the transition to motherhood for many mothers. Known as the antenatal period, this time is associated with challenging psychological, physiological, and social shifts (1, 2) and can lead to increased distress and psychological vulnerability (3, 4). Further, poor mental health during the antenatal period can also predict post-natal mental health issues faced by mothers (5–8). Despite its importance, mental health during pregnancy remains a relatively understudied part of the perinatal period especially in low and middle-income countries (LMIC) such as India.

Recent research suggests that the rates of perinatal depression, anxiety and suicidality are higher in LMICs, as compared to countries in the West (9–11). Possible explanations for these high rates are greater stigma attached to mental health care and a lack of awareness about perinatal mental health among practitioners and mothers, which subsequently impacts prevention and treatment of mental health concerns (12, 13). Furthermore, the current understanding of perinatal mental health in India is largely based on a medical model and symptom-focused approach (14–16). While these approaches have allowed researchers and practitioners to delineate mental illnesses specific to this period, and devise appropriate interventions (17–19), it seems necessary to add to this literature by expanding conceptions of mental health to include subjective experiences of mothers in the domains of psychosocial and individual factors.

Urban Indians face the unique combination of beliefs and lifestyle more aligned with the west, and the social and cultural practices of India (20). This amalgamation of traditional and modern views may impact mental health in the antenatal period. Thus, to understand the role of psychosocial factors on antenatal mental health in urban India, we examined how expecting mothers' perceptions of their attachment to the fetus, their sense of coherence, and perceptions of social support related to self-reported antenatal well-being and symptoms of antenatal depression.

Mothers' attachment to the fetus was studied by utilizing reports of maternal-fetal attachment. Maternal-fetal attachment has been found to be negatively associated with antenatal depression and positively associated with psychological well-being in Western samples (21–26). The study of maternal-fetal attachment among Indian samples has been surprisingly limited, and findings from these studies (27, 28) suggest that maternal-fetal attachment may play out differently in LMICs like India. There are a few possible explanations for this. Past research suggests that knowing the sex of the fetus is related to higher levels of maternal fetal attachment in the Western cultures (21, 29). However, sex determination is illegal in India, which may contribute to differences in the mother's attachment to the fetus. Further, women in India have historically played primarily care-taking roles. Although women in urban India have been joining the workforce in larger numbers over the last few decades, the stress of meeting familial expectations of gender roles has not necessarily reduced (30). In the 2016 IPSOS survey, 64% Indians believed that a woman's role is to be “a good mother and wife” (31). These expectations may lead to even greater levels of distress if the expecting mother does not develop strong maternal-fetal attachment. The current literature on maternal-fetal attachment is largely based on Western samples, and our understanding of how it is associated with antenatal mental health outcomes in urban India remains limited. We aim to address this gap by assessing the relationship between maternal-fetal attachment and antenatal mental health.

Next, to understand expecting mothers' experiences of social support from their partner, friends, and family, we examined their perceptions of social support. Evidence from several studies suggests that social support is particularly important for a woman's ability to cope during pregnancy (32–37). However, while social support was found to be a protective factor against poor antenatal mental health, this impact of social support is likely to vary based on culture and family context (38). Urban India presents a unique family context that amalgamates nuclear families with more traditional extended family settings. In larger families in India, social support may be more easily available which is beneficial for the expecting mother and fetus' health (39). Yet, poor relations with in-laws and partners can have the opposite effect and induce omnipresent stress (10, 40). For instance, in a cross-sectional study in Bihar, an Indian state, ill-treatment by in-laws was a predictor of antenatal depression (9). On the other hand, living in a nuclear family setting as compared to a more traditional extended household can also be a risk factor for poor antenatal mental health in urban India (10). In line with these findings, we sought to examine perceptions of social support and antenatal mental health in an urban Indian context.

Finally, we wanted to explore how expecting mothers make meaning of their experiences during pregnancy. Therefore, we studied expecting mothers' sense of coherence during pregnancy or their coping abilities and trust in positive outcomes in the face of antenatal challenges. According to Antonovsky, if one perceives stressful events as comprehensible, manageable, and meaningful, they may be less likely to experience depression and anxiety in the face of change, and report better health (41). Research has shown that a sense of coherence is associated with higher levels of antenatal well-being, uncomplicated deliveries (42, 43), better emotional health, and lower levels of anxiety, depression, and post-traumatic stress (44). Sense of coherence also seems to be a stronger predictor of antenatal well-being than social support in a Norwegian community sample (45). However, the association with antenatal mental health has not been investigated in LMICs and we hoped to initiate an understanding of sense of coherence alongside the study of perceived social support and maternal fetal attachment in urban India.

Through the current study, we aimed to address gaps in the literature on antenatal mental health in India. Specifically, we examined the cross-sectional associations of self-reported sense of coherence, perceived maternal-fetal attachment, and perceived social support with antenatal depression and well-being in a community sample of expecting mothers residing in urban India. We hypothesized that (a) maternal-fetal attachment will be positively correlated with antenatal well-being and negatively correlated with antenatal depression; (b) sense of coherence will be positively correlated with antenatal well-being and negatively correlated with antenatal depression; (c) social support will be positively correlated with antenatal well-being and negatively correlated with antenatal depression. In addition, we tested the statistically predictive value of models incorporating these psychosocial factors as predictors of antenatal depression and well-being.

## Methods

### Procedure

The study was cross-sectional in design. Data was collected entirely online through social media posts, with the questionnaire hosted on SurveyMonkey (46) between 23rd February 2020 and 9th March 2020. The social media posts included a link to the online questionnaire which took ~12 min to complete.

### Participants

Participants (*N* = 122; *M*_*age*_ = 28.07, *SD*_*age*_ = 3.72) were recruited using social media and directed to an online survey. Posts inviting first-time expectant mothers to participate were shared on Instagram, Facebook, WhatsApp, and online pregnancy forums. Inclusion criteria for the study required participants to be nulliparous women who resided in urban India, were in their second or third trimester of pregnancy, and fluent in English. We chose to exclude women in their first trimester of pregnancy as there is a greater risk of miscarriage, and Indian women prefer to disclose pregnancy in the second trimester.

Based on previous research on the psychosocial correlates of antenatal mental health (21, 24, 32–35, 42, 44), we expected a relationship of medium effect size (*f*^2^ = 0.20) among our predictor and outcome variables. An a-priori sample size calculation for multiple regression analysis using GPower (47) suggested that a sample of 81 participants is required to detect these effects at alpha level 0.01 with 0.80 statistical power.

### Materials

The following measures were included in the online questionnaire (see Table 1 for descriptive statistics).

**Table 1:** Summary of scales used to measure antenatal well-being, antenatal depression, perceived social support, sense of coherence and maternal-fetal attachment.

**Variable**	**Measure**	**Item range**	**Mean**	**Standard deviation**	**Skewness**	**Kurtosis**	**Reliability index (α)**
Antenatal well-being	SWEMWBS	1–5	3.375	0.6272	−0.4555	0.8559	0.783
Antenatal depression	EDS	0–3	1.152	0.5587	−0.003125	−0.4479	0.8444
Perceived social support	MSPSS	1–7	5.719	0.9253	−0.6484	0.2123	0.901
Sense of coherence	SOC13	1–7	4.335	1.058	0.3723	−0.3204	0.822
Maternal-fetal attachment	MFAS	1–5	3.960	0.4512	−0.1706	−0.3877	0.790

*SWEMWBS, Short Warwick Edinburgh Mental Well-being Scale; EDS, Edinburgh Depression Scale; MSPSS, Multidimensional Scale of Perceived Social Support; SOC13, Sense of Coherence Scale; MFA, Maternal Fetal Attachment Scale*.

#### Edinburgh Depression Scale

The Edinburgh Depression Scale (48) was used to measure antenatal depression. It has been validated for use during pregnancy (49). The scale consists of 10-items and had a high internal consistency α of 0.84 in our study.

#### Multidimensional Scale of Perceived Social Support

The 12-item Multidimensional Scale of Perceived Social Support (50) has three subscales to measure perceived social support from different sources, namely; family, friends, and partner. It has been used extensively in antenatal populations (51). In our study we found a high internal consistency (*α* = 0.90) for this measure.

#### Short Warwick Edinburgh Mental Well-Being Scale

Psychological well-being was measured using the Short Warwick Edinburgh Mental Well-Being Scale (52), a 7-item scale with a 5-point rating scale. The scale demonstrated high internal consistency (α = 0.78) when used in this study.

#### Maternal Fetal Attachment Scale

The Maternal Fetal Attachment Scale (53) is a 24-item self-report questionnaire. The original scale includes items specific to the third trimester so a shorter 17-item version (54), applicable to the second and third trimester was used. The scale had high internal consistency in the present study (α = 0.79).

#### Sense of Coherence Scale

The Sense of Coherence Scale-13 (55), is derived from the original 29-item version. It covers three primary components of sense of coherence; comprehensibility, manageability and meaningfulness. It has been used before in non-pregnant Indian populations (56) and also exhibited a high internal consistency (α = 0.822) in our study.

### Analysis

Data was analyzed using RStudio (57) and JASP 2.0 (58). Each demographic variable was measured against antenatal depression and well-being using bivariate correlation analysis. Relationships among perceived social support, sense of coherence, maternal-fetal attachment, antenatal depression and well-being were assessed using correlation analyses. Next, two regression models were tested to examine the contributions of maternal-fetal attachment, perceived social support, and sense of coherence entered together on reports of antenatal depression and well-being. Finally, sense of coherence was also tested as a mediator between social support and antenatal well-being as part of our exploratory analysis.

### Ethical Approval

The study was reviewed and approved by the Institutional Review Board, Ashoka University. The participants also provided informed consent to participate in this study.

## Results

### Participants

In our final sample, 54.9% participants were in their third trimester while 45.1% were in their second trimester. All but four participants were carrying a single child. All participants resided in cities in urban India with the majority of participants (69.6%) residing in Mumbai (23.0%), Bengaluru (18.0%), New Delhi (9.8%), Chennai (9.0%), Pune (4.9%), and Hyderabad (4.9%). All participants were married. When asked about a psychological illness, 87.7% participants reported no diagnosis before pregnancy, 9.8% participants reported having a psychological diagnosis before pregnancy, while 2.5% participants did not disclose this information; 91.8% participants reported no diagnosis of psychological illness during pregnancy, 5.7% participants reported having received a psychological diagnosis during, and 2.5% participants chose not to disclose this information. Sixty-eight percent of participants reported living in a joint family, while 32% percent of participants reported living in nuclear families. Lastly, 82.5% of participants reported being employed in the year prior to pregnancy, while 17.5% reported that they had not been employed.

We also collected information regarding pregnancy specific variables. The total time to conception ranged from no time (unplanned pregnancy) to 5 years (*M* = 8.06 months, SD = 13.46 months). A majority of participants (91%) reported a natural conception and 9% of participants reported a medically assisted conception. Seventy-eight percent participants reported no complications in the pregnancy, while 22.1% participants reported at least one complication. When asked about whether their doctor had spoken to them about antenatal mental health, 68.9% of participants reported that their doctor had not spoken to them about mental health during pregnancy. Finally, 79.5% participants reported not having experienced a miscarriage previously, 18.9% participants reported having experienced one miscarriage each, while two participants reported having experienced two and three miscarriages, respectively.

### Correlation Analyses

Maternal-fetal attachment was not significantly correlated with antenatal depression (*r* = −0.10, *p* = 0.286; see correlation matrix in Table 2), while it was positively related to antenatal well-being (*r* = 0.29, *p* = 0.001) partially supporting our hypothesis. In line with our hypothesis, sense of coherence was negatively associated with antenatal depression (*r* = −0.63, *p* < 0.001) and positively associated with well-being (*r* = 0.40, *p* < 0.001). Similarly, in line with our hypothesis, perceived social support was negatively correlated with antenatal depression (*r* = −0.51, *p* < 0.001) and positively related to well-being (*r* = 0.31, *p* < 0.001).

**Table 2:** Correlation Matrix - antenatal depression, antenatal well-being, maternal-fetal attachment, perceived social support, and sense of coherence.

**Variable**	**1**	**2**	**3**	**4**	**5**
Antenatal well-being	–				
Antenatal depression	0.44^**^	–			
Perceived social support	0.31^**^	−0.51^**^	–		
Sense of coherence	0.39^**^	−0.62^**^	0.51^**^	–	
Maternal-fetal attachment	0.29^*^	−0.10	0.17	0.10	–

*N = 122*;

* *p < 0.01*,

** *p < 0.001*.

We also checked if participant age and time since conception at participation were related to mental health. Age was not correlated with depressive symptoms (*r* = −0.01, *p* = 0.28) or well-being (*r* = 0.03, *p* = 0.76). Number of weeks since conception was not associated with antenatal depression (*r* = 0.17, *p* = 0.06) or well-being (*r* = −0.16, *p* = 0.08). Thus, we did not account for these variables in our regression models.

### Regression Analyses

We examined the combined contribution of our predictor variables on antenatal depression and well-being scores using two simultaneous regression models (Tables 3, 4). In the first regression model, maternal-fetal attachment, social support and sense of coherence were entered together in the first step, with antenatal depression scores as the outcome variables. The three variables together accounted for 42.5% variance in antenatal depression (Adjusted *R*^2^ = 0.425, *F*_(3,118)_ = 30.86, *p* < 0.001). In addition, only sense of coherence (*β* = −0.49, *p* < 0.001) and perceived social support (*β* = −0.25, *p* = 0.002) were found to uniquely contribute to the variance in antenatal depressive symptoms in this model.

**Table 3:** Model parameters for antenatal depression and well-being.

**Outcome**	***R* ^**2**^**	**Adjusted**	**dfs**	***F***	***p***
Antenatal depression	0.44	0.43	3, 118	30.86	<0.001
Antenatal well-being	0.23	0.21	3, 118	11.70	<0.001

**Table 4:** Coefficient estimates for regression models.

**Predictor**	**Unstandardized**		**Standardized**	***t***	***p***
	**B**	**SE**	***β***		
**DV: antenatal depression**					
Intercept	3.19	0.39		8.22	<0.001
Social support	−0.16	0.05	−0.26	−3.17	0.002
Sense of coherence	−0.26	0.04	−0.50	−6.19	<0.001
Maternal fetal attachment	−0.004	0.09	−0.003	−0.05	0.96
**DV: antenatal well-being**					
Intercept	0.83	0.51		1.64	0.11
Social support	0.07	0.06	0.11	1.51	0.25
Sense of coherence	0.19	0.06	0.32	3.42	<0.001
Maternal fetal attachment	0.33	0.11	0.24	2.86	0.005

In the second model, with maternal fetal attachment, social support and sense of coherence entered together in the first step, with antenatal well-being scores as the outcome variable, we found that the three variables accounted for 21% variance (Adjusted *R*^2^ = 0.21, *F*_(3,118)_ = 11.70, *p* < 0.001) in well-being scores. On further examination, we also noted that only sense of coherence (*β* = 0.32, *p* < 0.001) and maternal-fetal attachment (*β* = 0.24, *p* = 0.005) uniquely contributed to the variance in reports of antenatal well-being in this model.

### Exploratory Analyses

The correlations among the predictor variables were examined to understand how they relate to each other during the antenatal period. Maternal-fetal attachment was not correlated with either perceived social support (*r* = 0.174, *p* = 0.056) or sense of coherence (*r* = 0.10, *p* = 0.272). Perceived social support related positively to sense of coherence (*r* = 0.51, *p* < 0.001). We wondered if the relationship between perceived social support and antenatal well-being is mediated by expecting mothers' sense of coherence. To examine this relationship further, we conducted a mediation analysis using Baron and Kenny's (59) guidelines. Sense of coherence was analyzed as a mediator in the relationship between social support and well-being. Using regression analysis, we tested whether the effect of social support on antenatal well-being reduced or became non-significant if we added sense of coherence as a covariate. Results indicated that the contribution of social support was no longer significant when sense of coherence was added to the model, providing support for the mediating effect of sense of coherence in the relationship between social support and antenatal well-being (Adjusted *R*^2^ = 0.162, *F*_(2,119)_ = 12.68, *p* < 0.001). To assess the significance of the mediation effect, we used the bootstrap method as suggested by Preacher & Hayes (60) with 500 simulations on RStudio. Results indicated that the mediation effect is significant (*p* < 0.01).

We also examined associations between our variables of interest and demographic questions to make note of any relationships that can be further examined in future research. We analyzed if speaking to one's doctor was related to sense of coherence using a *t*-test and noted that women who reported that their doctors had spoken to them about antenatal mental health had higher levels of comprehensibility on the sense of coherence scale (*t* = −2.063, *p* = 0.041).

## Discussion

To our knowledge, this was the first investigation of maternal-fetal attachment, perceived social support and sense of coherence in relation with antenatal mental health in urban India. Our findings largely supported our hypotheses as our set of predictors contributed to 21% of the variance in antenatal well-being and 42.5% of the variance in symptoms of depression during pregnancy. Moreover, results from these regression models indicated that perceived social support and sense of coherence uniquely contributed to the variance in antenatal depressive symptoms, while maternal-fetal attachment and sense of coherence uniquely contributed to the variance in antenatal well-being. We discuss these findings in the following sections.

In the present study, maternal-fetal attachment was positively related to well-being and uniquely contributed to the variance in antenatal well-being scores. This finding is consistent with previous research (21, 22, 61, 62). However, contrary to our expectation, maternal-fetal attachment did not relate to antenatal depression. A potential explanation for this finding might pertain to the high mean score of maternal-fetal attachment (67.3 out of 85, SD = 7.6) in our non-clinical sample, with the lowest score being 48. Thus, in our sample most mothers reported feeling high levels of attachment to their fetus. These high levels might be a result of more traditional gender roles in India, contributing to women feeling more connected with the fetus, or obligated to give more socially desirable answers. On the other hand, maternal fetal attachment was positively related to expecting mothers' reports of well-being, suggesting that the more closely connected mothers felt to their fetus, the more likely they were to also experience better levels of well-being. Thus, it seems important to develop interventions to encourage maternal-fetal attachment among urban Indian women. A number of studies in countries like Iran and Korea have successfully used education-based approaches to increase maternal fetal attachment with a focus on topics like the importance of tactile stimulation, talking to the fetus, and father's role in maternal-fetal attachment (63, 64). There is already a prevalence of Lamaze classes in urban India, which can be modified to include awareness on perinatal mental health, maternal-fetal attachment and what to expect when the baby is born.

Next, in line with previous literature from western samples (42, 43) we found that sense of coherence uniquely contributed to both antenatal well-being and depression in models where perceived social support, sense of coherence, and maternal-fetal attachment were examined together. This suggests that despite being a relatively understudied factor in antenatal mental health in India, sense of coherence is associated with experiences of mental health during pregnancy. Results from our study also indicated a positive correlation between the comprehensibility subscale of sense of coherence and one's doctor talking to the woman about mental health during pregnancy. We also noted that only 31.1% of our participants reported that their doctor had spoken to them about antenatal mental health. Thus, a possible implication of these findings is the importance of training OBGYNS, physicians and nurses performing perinatal care in urban India to discuss mental health as well as general health concerns that may come up during pregnancy to help expecting mothers perceive their pregnancy as making sense, being predictable and explainable.

Next, we found that social support was associated with higher levels of well-being and lower levels of depression, consistent with the existing research (32–37). While social support uniquely contributed to antenatal depression in a model with sense of coherence and maternal-fetal attachment as predictors, we found that it did not uniquely contribute to antenatal well-being in a model with the same set of predictors. In other words, our findings point to the importance of the *absence* of perceived social support in contributing to depression, rather than its *presence* uniquely contributing to well-being in our regression models. Perhaps by virtue of being part of a collectivist culture, perceived social support is expected among pregnant women. Thus, receiving support from their families serves to only deter depressive symptoms, and is not perceived as additional, unexpected help that would promote antenatal well-being, in the presence of strong attachment to the fetus and higher levels of sense of coherence. This finding was contrary to our expectations based on previous reviews of the literature (10, 35, 65) and points to the importance of examining urban Indian women's unique family dynamics surrounding social support. It would be helpful for future studies to examine the relationship between social support and mental health factors among expecting mothers in urban India, while taking family structures into consideration. A current implication of the findings from the present study appears to be the importance of utilizing techniques to increase perceptions of social support among pregnant women to improve well-being. For example, health care facilities could create support groups for pregnant women, which will allow women who are experiencing a challenging time to connect with others having similar experiences (66).

Lastly, based on the positive correlation between social support and sense of coherence, as well as the unique contribution of sense of coherence on antenatal mental health, we theorized that expecting mothers' perception of high levels of social support may allow them to develop and maintain higher levels of sense of coherence, which could then contribute to better well-being. Our results provide some support for this claim. It appeared that the relationship between perceived social support and well-being was mediated through sense of coherence. However, this finding should be interpreted with some caution, as we assessed sense of coherence, perceptions of social support, and antenatal well-being through a cross-sectional design in this study and future research utilizing longitudinal design is needed to test these postulations further.

## Limitations and Conclusions

Our study is unique in its exploration of psychosocial factors that are associated with antenatal mental health among a sample of expecting mothers in urban India. Still, it is pertinent to take note of certain limitations of this study. First, the study used a cross-sectional correlation design, and thus we are not able to draw clear causal inferences among the variables studied. It might be possible that lower levels of depression and higher levels of well-being in expecting mothers could contribute to the development of a stronger maternal-fetal bond, greater sense of coherence and increased perceptions of social support. We have offered potential theoretical reasonings on the possible nature of the relationships among the variables in our discussion, but future research is needed to test these claims further. Second, our sample may be vulnerable to a self-selection bias. Women who found the study interesting or had greater psychological suffering may have been more likely to participate in this study. Similarly, most participants belonged to the upper-middle socio-economic strata. Thus, our sample addresses the experiences of a subset of expecting mothers in India and is not representative of the larger Indian population. Finally, data was collected using self-report measures and thus our findings reflect the subjective experiences of expecting mothers.

To conclude, this study is the first to examine sense of coherence, maternal-fetal attachment and perceived social support together terms of how they relate to antenatal well-being and depression among expecting mothers in India. An important implication of our findings is the need to tend to the ways in which expecting mothers view the world, feel an emotional bond with their unborn baby and experience social support in assessing and working with antenatal mental health concerns. Our results also indicate the importance of examining both well-being and depression as components of antenatal mental health, and the difference in results for these components suggests that we need to examine the psychosocial factors relating to both independently. Future research incorporating a sense of coherence, social support and maternal-fetal attachment in the context of antenatal mental health and well-being would continue to help in establishing guidelines for mental health care for expecting mothers.

## Data Availability Statement

The original contributions presented in the study are included in the article, further inquiries can be directed to the corresponding author.

## Ethics Statement

The studies involving human participants were reviewed and approved by Institutional Review Board, Ashoka University. The patients/participants provided their written informed consent to participate in this study.

## Author Contributions

Each author has made a unique contribution to this manuscript. MP and AB have been responsible for the conceptualization, design, and data collection of this study. MP, SK, AS, and AB have worked on the data analysis and interpretation, as well as the drafting and revision of the article. All authors have reviewed and approved this final version of the manuscript.

## Conflict of Interest

The authors declare that the research was conducted in the absence of any commercial or financial relationships that could be construed as a potential conflict of interest.

## Publisher's Note

All claims expressed in this article are solely those of the authors and do not necessarily represent those of their affiliated organizations, or those of the publisher, the editors and the reviewers. Any product that may be evaluated in this article, or claim that may be made by its manufacturer, is not guaranteed or endorsed by the publisher.
